# Supplementary material for: A sustainable ultra-high strength Fe18Mn3Ti maraging steel through controlled solute segregation and α-Mn nanoprecipitation
Source: Nat Commun. 2022 Apr 28;13:2330. doi: 10.1038/s41467-022-30019-x (PMC9050706; doi:10.1038/s41467-022-30019-x)
Supplement: Supplementary file 1 — Supplementary Information [file 41467_2022_30019_MOESM1_ESM.pdf]

Supplementary Information for:

A sustainable ultra-high strength Fe18Mn3Ti maraging steel through controlled solute segregation and α-Mn nanoprecipitation

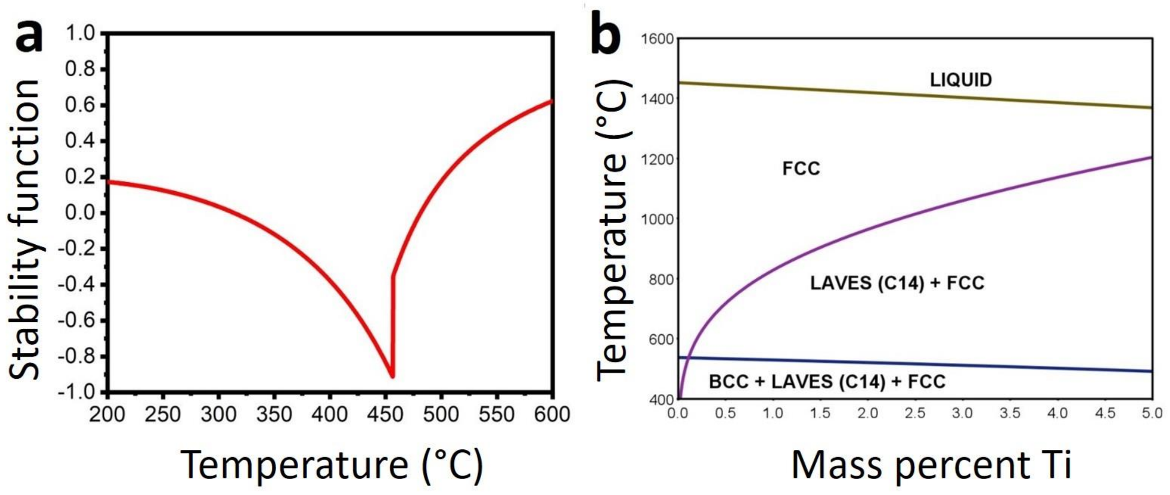

Supplementary Fig. 1: Thermodynamic calculations: a, normalized stability function of the BCC phase for the Fe18Mn3Ti alloy composition calculated using the TCFE9 thermodynamic database. **b**, pseudo-binary equilibrium phase diagram for the Fe-18Mn-xTi (wt.%) composition calculated using the HMnS04 database.

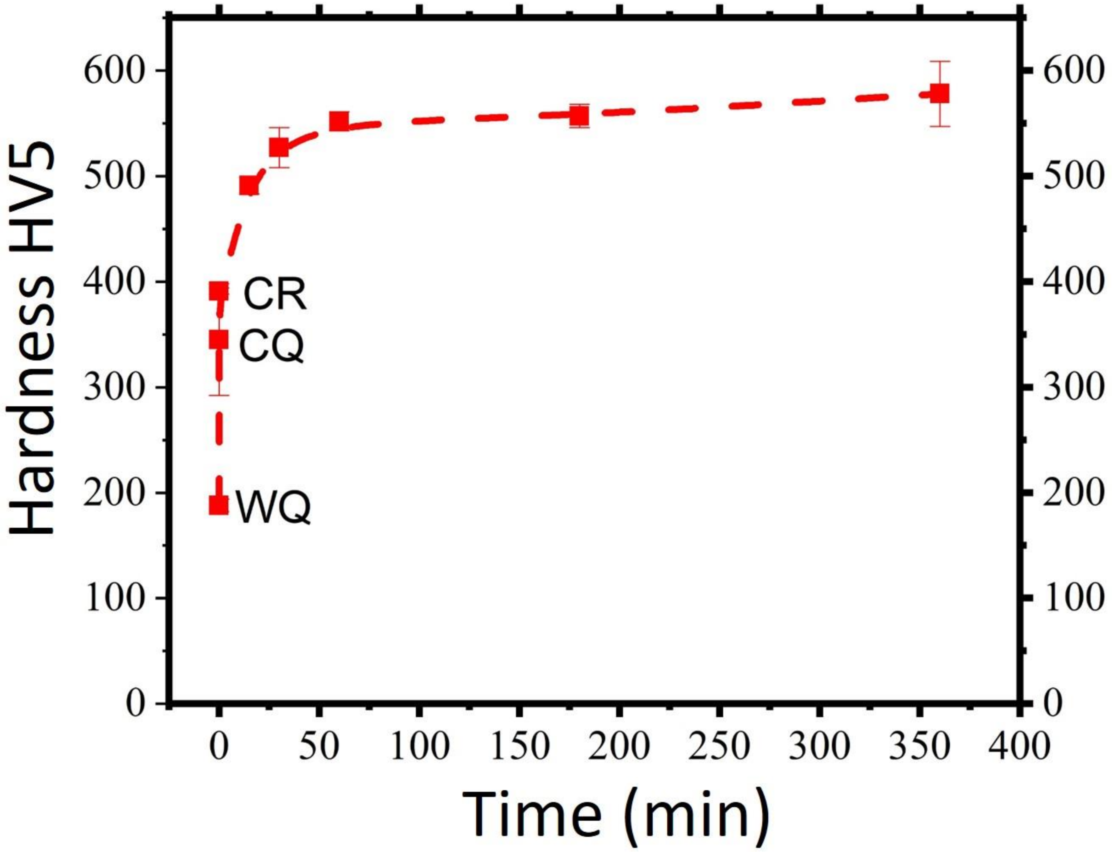

Supplementary Fig. 2: Hardness measurements: Vickers hardness (HV5) of the Fe18Mn3Ti alloy water quenched (WQ), cryo quenched (CQ), cold rolled (CR) and annealed up to 6h at 450 ºC.

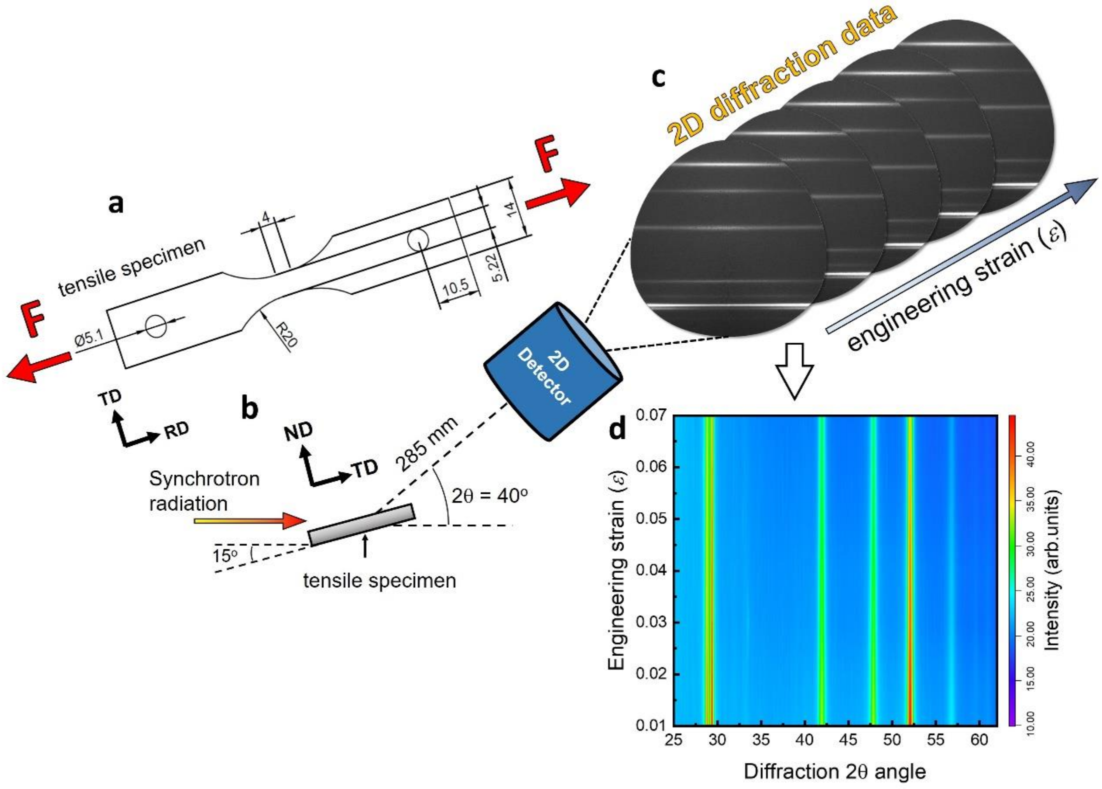

Supplementary Fig. 3: In-situ synchrotron XRD: (a) schematic representation of the tensile specimen used for tensile testing in conjunction with in-situ synchrotron XRD. TD and RD represent the transversal and rolling direction of the original plate, respectively. **(b)** Schematic representation of the experimental geometry used at the XTMS workstation. **(c)** 2D diffraction data collected over tensile testing. **(d)** Evolution of the 'intensity versus 2θ diffraction angle' plots during tensile testing.

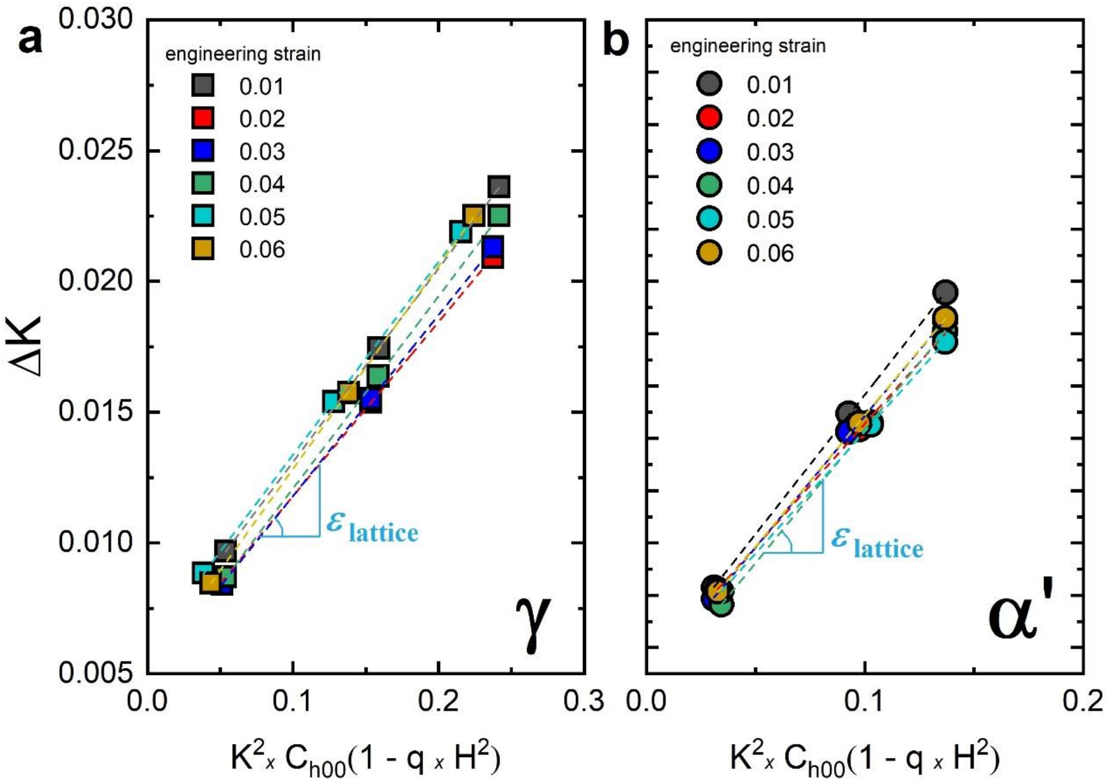

Supplementary Fig. 4: Modified Williamson-Hall plots: (a) for austenite and **(b)** α'-martensite deformed at representative engineering strains.

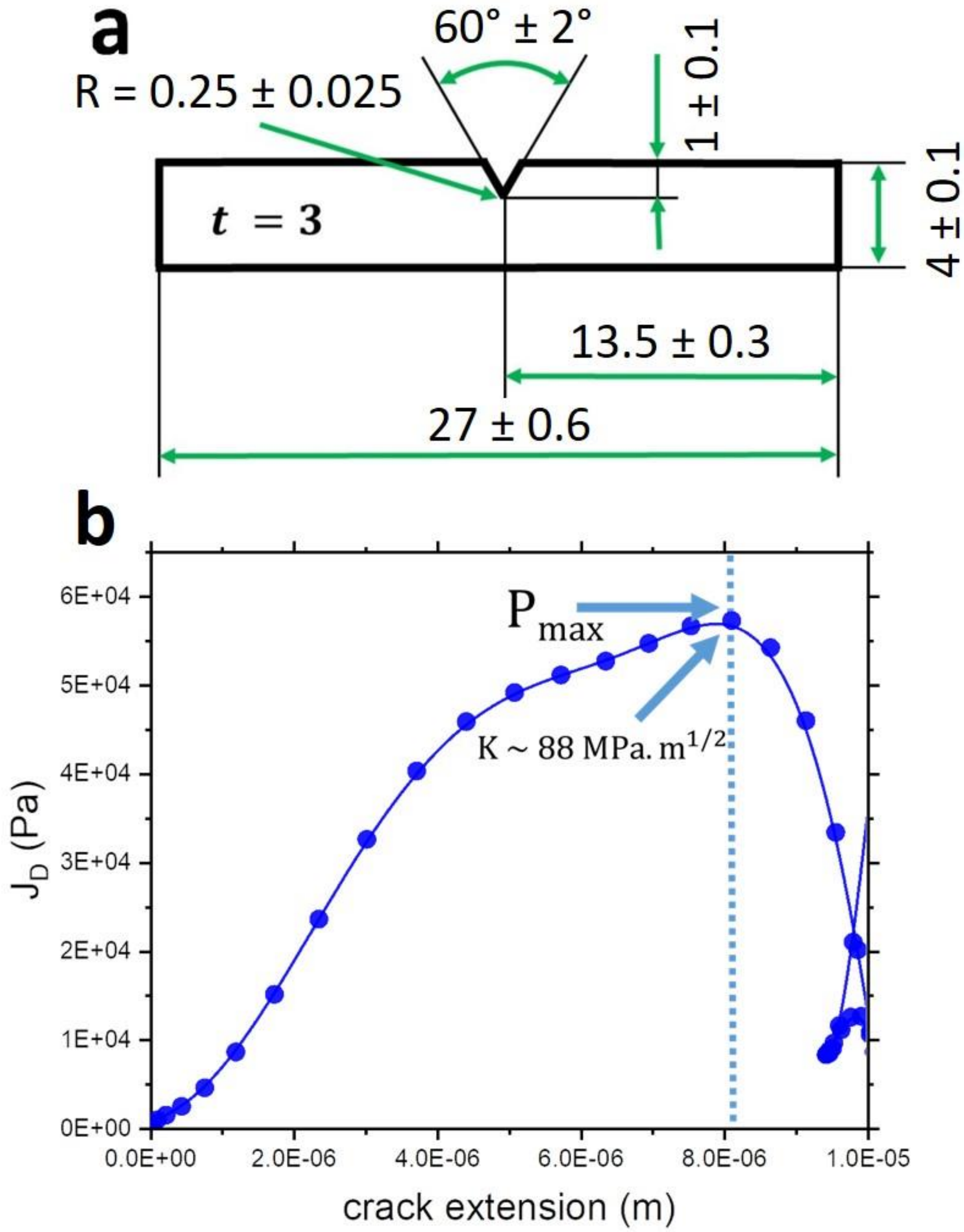

Supplementary Fig. 5: Impact testing: (a) Geometry of the sub-sized Charpy V-notched (CVN) samples. (b) Dynamic J-integral curve obtained from room temperature (25°C) instrumented impact testing of the Fe18Mn3Ti alloy aged during 1h@450°C.

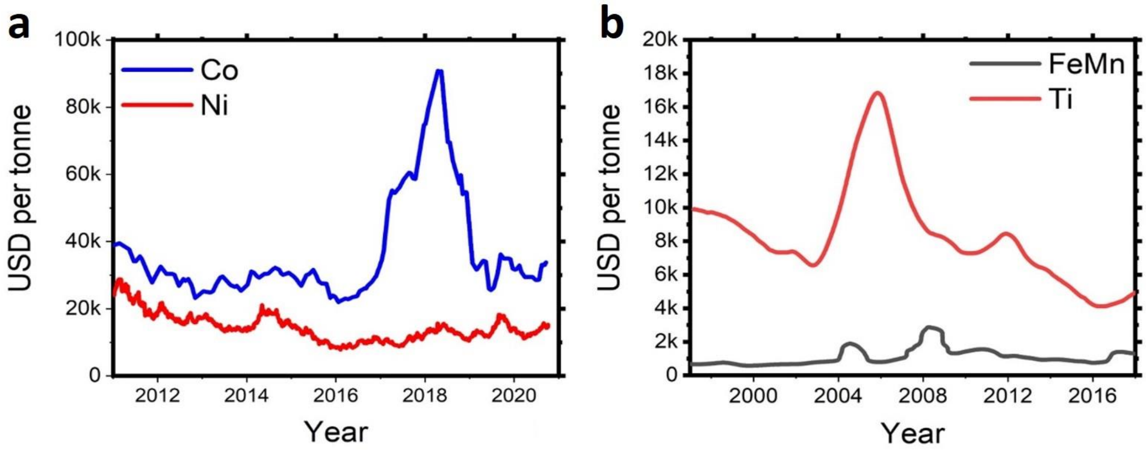

Supplementary Fig. 6: Fluctuation of the cost of different alloying elements: (a) LME cobalt and nickel historical price. (b) Historical price of titanium and ferromanganese (FeMn). Smoothed data.

Supplementary Table 1 Chemical composition of Fe18Mn3Ti alloy in wt.% according to wet-chemical analysis

Mn	Ti	Al	C	N	Ni	O	Fe
17.6	2.84	<0.002	0.0080	<0.002	0.0038	0.0050	Balance

Supplementary Table 2 – Chemical composition in wt.% of the different alloys used for comparison in this work.

Alloy	Fe	Cr	Ni	Co	Mo	Ti	Al	Mn	Cu
**18Ni(200)**	69.9		18	8.5	3.3	0.2	0.1		
**18Ni(250)**	68		18	8.5	5	0.4	0.1		
**18Ni(300)**	67.2		18	9	5	0.7	0.1		
**18Ni(350)**	63.6		18	12.5	4.2	1.6	0.1		
**Co-free 18Ni(250)**	77		18.5		3	1.4	0.1		
**Low-Co 18Ni(250)**	75.6		18.5	2	2.6	1.2	0.1		
**PH17-4**	79.3	16.2	3.9					0.5	3.4
**PH13–8Mo**	75.8	12.7	8.2		2.2		1.1		
**FeCoNiMnCr**	19.91	18.54	20.93	21.01				19.59	
**NiCoCr**		30.65	34.6	34.74					
